# TanhReLU -based convolutional neural networks for MDD classification

**DOI:** 10.3389/fpsyt.2024.1346838

**Published:** 2024-05-31

**Authors:** Qiao Zhou, Sheng Sun, Shuo Wang, Ping Jiang

**Affiliations:** ^1^ Computer School (Huangshi Key Laboratory of Computational Neuroscience and Brain-Inspired Intelligence), Hubei Polytechnic University, Huangshi, China; ^2^ Electronic information and electrical engineering institute, Hubei Polytechnic University, Huangshi, China

**Keywords:** classification, major depression disorder (MDD), EEG, TanhReLU, CNN

## Abstract

Major Depression Disorder (MDD), a complex mental health disorder, poses significant challenges in accurate diagnosis. In addressing the issue of gradient vanishing in the classification of MDD using current data-driven electroencephalogram (EEG) data, this study introduces a TanhReLU-based Convolutional Neural Network (CNN). By integrating the TanhReLU activation function, which combines the characteristics of the hyperbolic tangent (Tanh) and rectified linear unit (ReLU) activations, the model aims to improve performance in identifying patterns associated with MDD while alleviating the issue of model overfitting and gradient vanishing. Experimental results demonstrate promising outcomes in the task of MDD classification upon the publicly available EEG data, suggesting potential clinical applications.

## 1 Introduction

Major Depression Disorder (MDD), characterized as a multifaceted mental health disorder, poses formidable challenges in achieving precise and reliable diagnostic outcomes (1). Accurate classification of MDD is crucial for effective treatment and personalized interventions. In contrast to subjective measurement tools such as electroencephalogram (EEG), functional magnetic resonance imaging (fMRI) (2, 3), and computed tomography (CT) present notable advantages in terms of objectivity within the domain of MDD diagnosis. Of particular interest among these, EEG is characterized by (1) exceptional temporal resolution, enabling the real-time capture of neural activity, and (2) a relatively heightened cost-effectiveness in comparison to both fMRI and CT. This cost efficiency renders EEG a pragmatic choice for both research endeavors and clinical applications (4), thus garnering substantial attention from the scholarly community.

The burgeoning field of data-driven approaches (5), particularly the utilization of EEG data, holds promise in enhancing diagnostic accuracy. Specifically, the paradigm of deep learning has emerged as a potent tool for unraveling intricate patterns inherent in the complex domain of MDD. For example, in the early stage of MDD classification, in pursuit of leveraging EEG signals for nonlinear analysis and subsequent discrimination between individuals with MDD and a control cohort, Hosseinifard et al. undertook the extraction of power spectra from four EEG frequency bands along with four distinct nonlinear features. The discriminative task involved the application of classifiers, including k-nearest neighbors, linear discriminant analysis, and logistic regression, to differentiate between 45 unmedicated individuals with MDD and 45 demographically matched controls and achieved a classification accuracy of 83.3% (6). From then on, Rajendra et al. employed Convolutional Neural Networks (CNNs) for the screening of MDD based on EEG signals. The CNN model autonomously adapts and learns discriminative features from input EEG signals, distinguishing between EEGs originating from individuals with MDD and those from normal subjects. Experimental evaluation, conducted on EEG data from 15 normal individuals and 15 patients with MDD, yielded classification accuracies of 93.5% and 96.0%, respectively (7). Recently, in the pursuit of monitoring mental MDD through EEG data, a comparative analysis involving four neural network-based deep learning architectures (MLP, CNN, RNN, RNN with LSTM) and two Supervised Machine Learning Techniques (SVM and LR) was conducted. The experimental findings pertaining to the classification performance in discerning the presence of mental MDD from EEG data reveal an intriguing outcome—the classification performance of SVM surpassed that of deep learning methodologies (8). Nowadays, Chen et al. introduce DCLNet, a short time series model based on CNN, designed for the classification of MDD. While DCLNet relies on conventional signal preprocessing procedures, it demonstrates outstanding performance in the task of MDD classification (9).

Despite the outstanding performance of deep learning in MDD classification, the vulnerability to overfitting in these models presents a significant challenge that warrants careful consideration. In the realm of mitigating model overfitting, predominant strategies encompass data augmentation, regularization, feature selection, and the reconfiguration of activation functions, among others, each exhibiting distinct advantages and drawbacks across diverse domains. Data augmentation, exemplified by its capacity to enhance data diversity, contributes to heightened model generalization, albeit with the potential introduction of noise (10). Regularization, through the introduction of penalty terms to curtail model parameter magnitudes, mitigates overfitting risks Santos and Papa (11). However, the optimal regularization strength necessitates careful tuning, as excessive regularization may overly simplify the model, impairing its ability to capture intricate patterns. Feature selection, a technique for eliminating redundant or irrelevant features, streamlines models and reduces overfitting potential, yet the issue of selection remains a quintessential NP-hard problem (12). These methodologies collectively underscore the nuanced landscape of overfitting alleviation, demanding meticulous consideration of their applicability and trade-offs within specific contexts. The phenomenon of gradient vanishing can adversely impact the efficacy of model training, preventing the model from adequately learning the features of the data. This, in turn, can diminish the model’s generalization capability and increase the risk of overfitting. Therefore, addressing the issue of gradient vanishing can enhance the efficiency and quality of model training, thereby reducing the likelihood of overfitting.

To this end, this study endeavors to address the overfitting and gradient vanishing challenge in the classification of MDD by introducing a novel approach: a TanhReLU-based Convolutional Neural Network (CNN). The TanhReLU activation function, amalgamating the advantageous characteristics of Tanh and rectified linear unit (ReLU) activations, is employed to augment the model’s capability in recognizing intricate patterns associated with MDD. The primary objective is not only to improve classification performance but also to mitigate the common issue of overfitting, thereby enhancing the model’s generalization to new data. Thus, the contributes are summarized as below:

1. This study addresses the challenge of gradient vanishing by introducing a TanhReLU-based CNN by combining characteristics of Tanh and ReLU activations, to enhance MDD pattern identification and mitigate issues of overfitting.
2. Experimental results on publicly available EEG data demonstrate promising outcomes in MDD classification, indicating potential clinical applications for the proposed approach.

In summary, this study introduces a TanhReLU-based CNN to address the challenge of accurate diagnosis of MDD using EEG data. By integrating the TanhReLU activation function, the model aims to improve performance in identifying MDD-related patterns while mitigating issues such as model overfitting and gradient vanishing.

In the subsequent sections, we delve into the intricacies of our proposed TanhReLU-based CNN, elucidating its architectural nuances and the rationale behind the integration of the TanhReLU activation function. The study’s findings, rooted in comprehensive experimentation using publicly available EEG data, are presented to underscore the promising outcomes in MDD classification. These results, in turn, suggest potential applications of our proposed model in clinical settings, thereby contributing to the advancement of accurate and data-driven approaches to MDD diagnosis.

## 2 Methodology

This section initially discusses the design of TanhReLU, followed by an introduction to the design of a TanhReLU-based CNN. Finally, the training process of the TanhReLU-based CNN is described.

### 2.1 TanhReLU activation function

The use of saturating activation functions, such as Sigmoid or Tanh, in neural networks can lead to the problem of gradient vanishing, especially in deep neural networks. This is because when the input values are too large or too small, the derivatives of these activation functions approach zero, resulting in the attenuation of the error gradients during backpropagation. This can hamper the learning process and the convergence of the model parameters. Despite these drawbacks, Tanh function has several advantages as an activation function in neural networks. First, it is a smooth and differentiable function, which allows the use of gradient-based optimization algorithms. Second, it maps the input values to a bounded interval of (-1,1), which helps to regulate the output range and prevent numerical issues. Third, it introduces a nonlinear transformation, which enhances the expressive power of the neural network. Fourth, it has a symmetric output range, which reflects fairness. The Tanh function is also closer to the identity function than the sigmoid function, which can facilitate the convergence of the neural network.

Balancing the issues of gradient vanishing and gradient exploding is a crucial challenge in deep learning models. Currently, the main solutions include using ReLU activation function (13), batch normalization (14) and gradient clipping (15). However, these methods have their own advantages and disadvantages. For example, the effect of batch normalization depends on the choice of batch size, and gradient clipping may alter the direction of the original gradient, thus affecting the learning process of the model. ReLU activation function performs well in computational efficiency, but suffers from the so-called “dying ReLU” problem, that is, when the input is zero or negative, the gradient of ReLU becomes zero, resulting in the inability of the network to perform backpropagation. These problems suggest that we need more careful and comprehensive considerations when designing and optimizing deep learning models.

**Figure 1:**
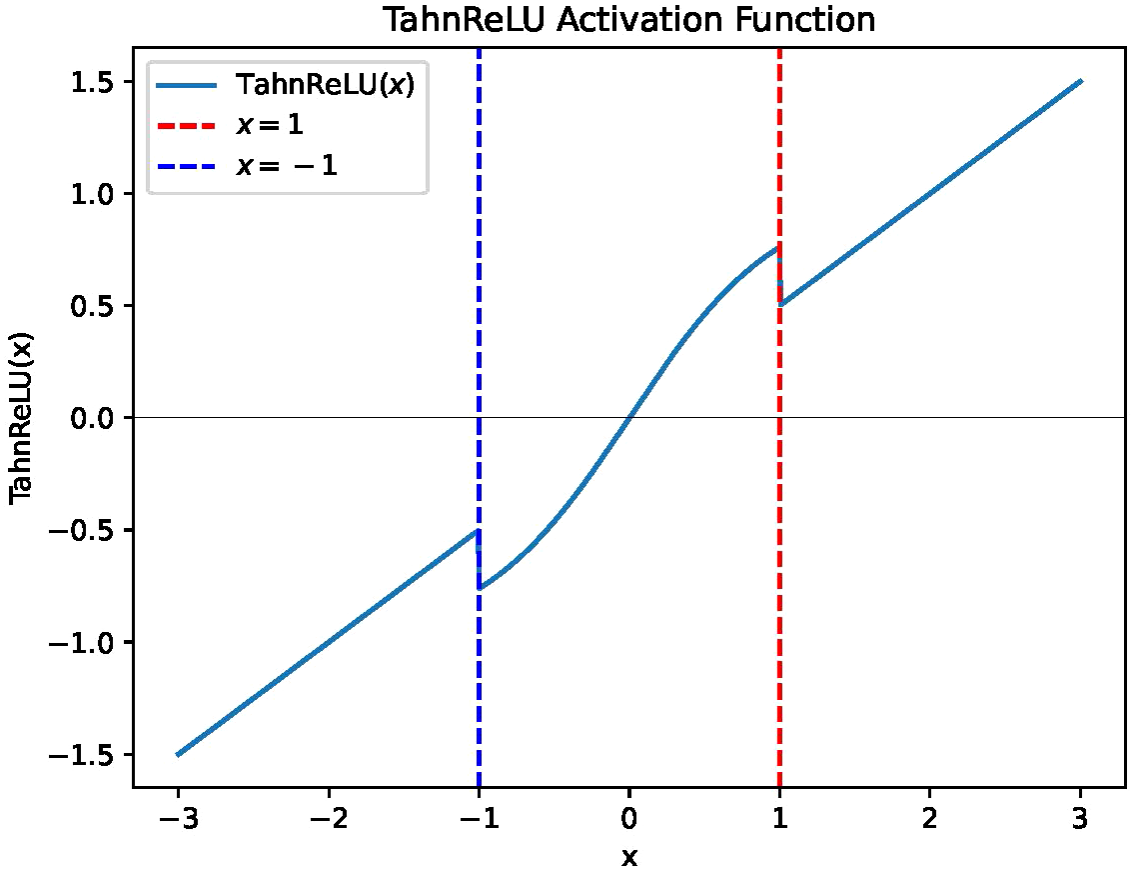
The graphical representation of TanhReLU with segmentation parameter of 1.


A natural motivation for addressing the drawbacks of Tanh and ReLU activation functions is to combine them in a hybrid way, which can eliminate the saturation problem of Tanh in the tails and the problem of “dying ReLU”. The graphical representation of the function is depicted in **Figure 1**. The function is defined as follows:

(1) $$\text{TanhReLU }(\text{x})=\{\begin{array}{ll} \frac{e^{x}-e^{-x}}{e^{x}+e^{-x}}, & \;\left|\text{x}\right|\leq a\\ \;\;0.5\text{x}, & \;\left|\text{x}\right|>a \end{array}$$

where the *a* is the segmentation parameter for the fused activation function (a = 0.25 in this study).

The graphical representation elucidates that Equation (1) exhibits a non-linear profile, indicative of its capacity for capturing intricate patterns and features. Notably, within the proximity of zero, the function manifests a smooth transition, akin to the characteristics observed in the tanh function. This attribute facilitates stable gradient propagation, thereby mitigating the issue of gradient vanishing. Moreover, for input values exceeding 1, the TanhReLU function mirrors the behavior of the conventional ReLU function, directly returning the input value. This retention of ReLU characteristics, including sparse activation and computational simplification, serves to alleviate the long-tail gradient vanishing problem. Importantly, the function avoids “dead ReLU” issues by ensuring non-zero values in the negative range. The graphical representation showcases symmetry about the origin (0, 0), highlighting fairness in its behavior. These characteristics collectively underscore the potential advantages of the TanhReLU function in neural network applications, particularly in addressing challenges associated with gradient vanishing and ensuring robust activation patterns.

### 2.2 Architecture of TanhReLU-based CNN

**Figure 2:**
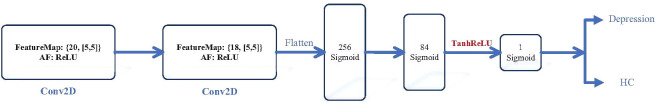
The graphical representation of the TanhReLU-based CNN.

**Figure 2** delineates the architectural framework of the TanhReLU-based Convolutional Neural Network (CNN), strategically designed to maximize classification accuracy with a minimized layer count. The CNN commences with dual convolutional layers employing identical receptive maps (5 × 5) followed by three fully connected (FC) layers. The figure provides a visual representation of the activation functions employed in each layer. Culminating in the Sigmoid activation function, the CNN produces conclusive outcomes for the identification of specific EEG segments. The salient features of this design are succinctly encapsulated as follows.

The objective of the “high-filter convolutional layer” is to engage with high-dimensional raw EEG segments by strategically deploying a substantial number of convolutional filters (20) within a singular convolutional layer. Each filter within this configuration is specifically tasked with processing data from an individual channel. Within the context of each temporal window, the time series data (1024) originating from each electrode undergoes reshaping into a square matrix format (32 × 32). Subsequently, the entire EEG segment is systematically structured into a three-dimensional data block, cascading along channels. This architectural design aims to enhance the network’s capacity for discerning intricate patterns within the EEG data.

The objective of the “Hourglass” FC layer block (16) is to expediently diminish the number of neurons, thereby curtailing the overall count of model parameters. This block encompasses multiple FC layers, with a diminishing number of neurons as one approaches the output layer. In the current study, the “Hourglass” fully connected layer block corresponds to the terminal three FC layers.

### 2.3 Training of TanhReLU-based CNN

The streamlined TanhReLU-based CNN undergoes training utilizing Stochastic Gradient Descent (SGD). A weight decay of 1e-6 is employed to maintain a low training error for the model. Weight initialization and batch normalization is applied across the network. Following the shuffling of the complete sample space, it is partitioned into training sets, validation sets, and test sets. A Leave-One-Out validation algorithm is employed to assess the training performance of the classifier using training and test sets, which is evaluated to report the classification performance. The weights and biases of the TanhReLU-based CNN are then trained utilizing the backpropagation algorithm. The primary objective of this optimizer is the minimization of the Mean Squared Error loss function, achieved by employing a learning rate of 0.01, thereby contributing to the overall enhancement of the model’s performance. The optimization process involves iteratively refining the model’s parameters through the Backpropagation algorithm. This iterative training process continues until a predefined termination criterion is met, specifically at Epoch 40, employing a batch size of 15, and incorporating early termination with a Patience value of 10.

This optimizer’s objective is the minimization of the Mean Squared Error loss function with a learning rate of 0.01, consequently contributing to the enhancement of the model’s performance. This optimization endeavor is facilitated through the continual refinement of the model’s parameters, a feat achieved via the Backpropagation algorithm. This iterative process of training persists until a specified termination criterion is satisfied (notably, at Epoch 40, with a batch size of 15, and early termination employing a Patience of 10). Upon the culmination of the training phase, an evaluation of the model’s efficacy is conducted through its deployment on an autonomous testing dataset. This assessment offers an impartial estimation of the model’s capacity to generalize to hitherto unseen data instances.

## 3 Results

The experiments conducted in this section serve as a validation and assessment of the classification performance of the proposed model. Initially, we introduce the dataset (refer to Section 3.1) after describing the experimental platform utilized for these assessments. Finally, the classification effectiveness of the TanhReLU-based Convolutional Neural Network (CNN) is evaluated using metrics such as accuracy, sensitivity, and specificity (see Section 3.2). The experiments were executed on a desktop equipped with an Intel i7 CPU operating at 3.33GHz, an Nvidia RTX 1080Ti GPU, 32GB RAM, and running Windows 10. This system configuration ensured consistent testing conditions throughout the experiments.

### 3.1 Dataset

The MPHC EEG Data Mumtaz et al. (17) were obtained from 34 Major Depressive Disorder (MDD) patients, including 17 males with a mean age of 40.3 ± 12.9, and 30 healthy subjects (the control group), comprising 21 males with a mean age of 38.227 ± 15.64. The data collection took place at the hospital of University Sains Malaysia. Exclusion criteria for MDD participants encompassed those with psychotic symptoms, pregnant individuals, alcoholics, smokers, and patients with epileptic problems. The healthy control group underwent screening for potential mental or physical illnesses and was confirmed to be disease-free. EEG sensors, following the 10–20 international system, were positioned on the scalp with 20 electrodes (Fp1, Fp2, F3, F4, F7, T3, T5, C3, C4, Fz, Cz, Pz, F8, T4, T6, P3, P4, O1, O2, ECG) at a sampling rate of 256 Hz. The time window size was set to 1024 (4 seconds), resulting in a total sample space of 18,442 segments (MDD: 9789, HC: 8653).

### 3.2 Performance on identifying MDD

**Figure 3:**
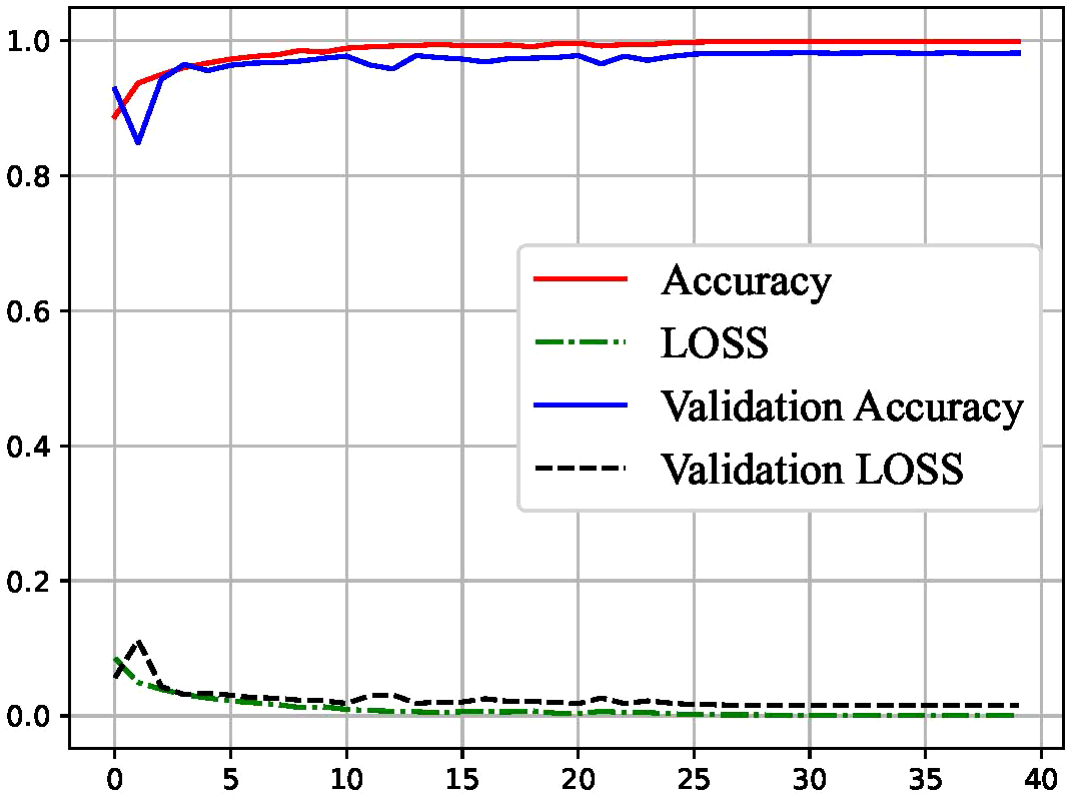
Learning Curve for classifying MDD.

For monitoring the training process, learning curves were employed. The results, as illustrated in **Figure 3**, reveal that our classifier underwent stable learning, demonstrating the absence of overfitting or underfitting. This observation suggests the generalizability of our approach. Moreover, the classifier exhibited exceptional performance on the test set, indicating its robust discriminative power in identifying MDD.

Finally, the model’s classification efficacy was evaluated on the designated test set. Our proposed approach achieved a remarkable accuracy of 98.59%, sensitivity of 98.77%, and specificity of 98.38%, as outlined in **Table 1**. A comparative analysis with foundational classifiers, including the multivariate logistic regression classifier-based wavelet (MLRW) (17), Resnet-16 (18), and CapsuleNet (19), demonstrates a notable enhancement in performance with our proposed methodology. Moreover, we conducted comparative analyses between TanhReLU and mainstream activation functions such as ReLU, Tanh, etc. This comparative study in **Table 1** will enable us to better understand the relative performance of TanhReLU and its suitability for MDD classification tasks. From the table, it can be observed that TanhReLU exhibits a slight advantage over ReLU and Tanh activation functions.

**Table 1:** Comparative analysis of the performance between the proposed methodology and existing state-of-the-art approaches.

Approach	Sensitivity (%)	Specificity (%)	Accuracy (%)
CapsuleNet	89.01	99.23	94.42
Resnet-16	88.9	74.79	82.26
MLRW	95	80	87.5
Our (Tahn)	98.46	98.16	98.32
Our (ReLU)	98.92	97.87	98.43
Our (TanhReLU)	98.77	98.39	98.59

## 4 Discussions and conclusions

### 4.1 Generalizability and stability of the classifier

The learning curves presented in **Figure 3** underscore the stability and generalizability of our classifier during the training process. The absence of overfitting or underfitting is crucial for ensuring that the model performs well not only on the training data but also on unseen test data. The classifier’s ability to maintain stable learning patterns is indicative of its potential applicability to diverse datasets and scenarios, bolstering its reliability in real-world applications.

### 4.2 Implications for personalized medicine

The success of our classifier in discerning MDD holds promise for personalized medicine in the context of MDD. Tailoring classifier approaches based on individual MDD is a longstanding goal in mental health care. The discriminative power demonstrated by our classifier suggests that it could contribute to a more nuanced and personalized understanding of MDD, potentially guiding clinicians in optimizing therapeutic strategies for individuals.

### 4.3 Limitations and future directions

While our study yields promising results, it is essential to acknowledge certain limitations. The dataset’s size and heterogeneity may impact the model’s generalizability to broader populations. Future research endeavors should involve larger and more diverse datasets to further validate and refine the classifier’s performance. Additionally, incorporating longitudinal data could enhance the model’s ability to capture dynamic changes in MDD severity over time.

Moving forward, future research could also explore additional enhancements to the CNN architecture, such as incorporating attention mechanisms or multimodal data fusion, to further improve classification performance. Furthermore, the application of this method could extend beyond MDD diagnosis, with potential utility in other medical domains such as anxiety disorders or neurodegenerative diseases.

### 4.4 Conclusions

This study introduces a TanhReLU-based CNN for EEG-based classification of MDD. The model effectively distinguishes between depressed and non-depressed individuals, as evidenced by rigorous evaluation. The integration of TanhReLU activation enhances the model’s ability to capture complex EEG patterns. Learning curves indicate stable training, ensuring generalizability. Evaluation on a separate test set yields impressive accuracy (98.59%), sensitivity (98.77%), and specificity (98.38%). Comparative analysis highlights the superior performance of the TanhReLU-based CNN. In conclusion, our model offers a robust and generalizable approach for MDD classification, with potential implications for personalized medicine and mental health care advancement. Further research is needed to fully explore its clinical utility.

## Data availability statement

The original contributions presented in the study are included in the article/supplementary material. Further inquiries can be directed to the corresponding author.

## Author contributions

QZ: Conceptualization, Project administration, Writing – original draft. SS: Investigation, Validation, Writing – review & editing. SW: Data curation, Methodology, Visualization, Writing – review & editing. PJ: Funding acquisition, Writing – review & editing, Validation.
